# Supplementary material for: Oxytetracycline Persistence in Uterine Secretion after Intrauterine Administration in Cows with Metritis
Source: Animals (Basel). 2022 Jul 28;12(15):1922. doi: 10.3390/ani12151922 (PMC9367300; doi:10.3390/ani12151922)
Supplement: Supplementary file 1 [file animals-12-01922-s001.zip › animals-1815219-SI.pdf]

Table S1. Measured individual concentrations of oxytetracycline in plasma and milk ($n = 6$) after the third intrauterine dose (8 mg/kg) of 10% solution of oxytetracycline.

**Time (h)**	**Cow 1 (μgmL^{-1})**	**Cow 2 (μgmL^{-1})**	**Cow 3 (μgmL^{-1})**	**Cow 4 (μgmL^{-1})**	**Cow 5 (μgmL^{-1})**	**Cow 6 (μgmL^{-1})**
Plasma						
0.5	0.057	<LOQ	0.091	0.074	0.277	0.038
1	0.051	0.022	0.098	0.066	0.163	0.101
3	0.044	<LOQ	0.109	0.063	0.098	0.058
6	0.039	<LOQ	0.089	0.052	0.084	0.056
9	0.033	<LOQ	0.080	0.046	0.077	0.106
12	0.029	<LOQ	0.054	0.043	0.070	0.072
24	0.022	<LOQ	0.046	0.038	0.065	0.037
48	<LOQ	<LOQ	0.025	<LOQ	<LOQ	0.024
72	<LOQ	<LOQ	<LOQ	<LOQ	<LOQ	<LOQ
96	<LOQ	<LOQ	<LOQ	<LOQ	<LOQ	<LOQ
Milk						
0.5	0.149	<LOQ	1.120	0.273	0.464	0.193
1	0.149	<LOQ	0.921	0.396	0.351	0.186
3	0.175	<LOQ	0.736	0.901	0.275	0.235
6	0.248	<LOQ	0.788	0.711	0.450	0.438
9	0.228	0.035	1.175	0.313	0.452	0.243
12	0.275	0.037	1.292	0.442	0.453	0.275
24	0.135	<LOQ	0.879	0.135	0.391	0.237
48	0.175	<LOQ	0.483	0.057	0.293	0.146
72	0.093	<LOQ	0.324	0.062	0.115	0.066
96	0.034	<LOQ	0.176	0.047	0.072	0.052

<LOQ – below limit of quantification.
